# Rethinking Postoperative Pain in the Era of Robotic Thoracic Surgery: A Surgeon’s Perspective on Functional Recovery and Potential Oncologic Implications

**DOI:** 10.3390/jcm15207843

**Published:** 2026-10-12

**Authors:** Takuro Miyazaki, Ryoichiro Doi, Keitaro Matsumoto

**Affiliations:** Division of Thoracic and Pelvic Surgery, Department of Surgery, Nagasaki University Graduate School of Biomedical Sciences, Nagasaki 852-8501, Japan; rdoi@nagasaki-u.ac.jp (R.D.); kmatsumo@nagasaki-u.ac.jp (K.M.)

**Keywords:** robot-assisted thoracic surgery, video-assisted thoracoscopic surgery, chronic postsurgical pain, intercostal nerve injury, Return to Intended Oncologic Treatment, minimally invasive thoracic surgery, lung cancer surgery

## Abstract

Persistent postoperative pain remains an incompletely resolved problem after minimally invasive thoracic surgery, despite expectations that robot-assisted thoracic surgery (RATS) would meaningfully outperform video-assisted thoracoscopic surgery (VATS) in analgesia. This narrative review synthesizes evidence from randomized trials, cohort studies, and meta-analyses comparing acute and chronic pain outcomes between VATS and RATS, examines mechanisms and surgically modifiable risk factors underlying intercostal nerve injury and pain chronification, and evaluates the emerging concept of Return to Intended Oncologic Treatment (RIOT) as a perioperative endpoint linking pain control to oncologic recovery. Across randomized and propensity-matched comparisons, RATS has generally failed to demonstrate superior pain outcomes relative to VATS, and several studies suggest multiport robotic platforms may produce greater early pain than uniportal thoracoscopic approaches. Port number, operative time, and drainage duration emerge as modifiable surgical predictors of chronic pain, while inadequately controlled acute pain compromises functional recovery and is associated with delayed RIOT; direct evidence that pain independently influences RIOT or long-term survival remains limited, and this link should currently be regarded as a plausible, testable hypothesis rather than an established causal pathway. We conclude that, on the comparative evidence available to date, robotic instrumentation has not shown a consistent, clinically meaningful reduction in pain burden relative to VATS, and that pain management should be reframed as a shared surgical-anesthetic target relevant to oncologic recovery rather than a comfort-oriented endpoint alone. Emerging robotic platforms warrant evaluation against this expanded outcome set.

## 1. Introduction

Minimally invasive surgery has become the default approach for anatomic lung resection, yet persistent postoperative pain has not disappeared along with the large posterolateral incision. Video-assisted thoracoscopic surgery (VATS) markedly reduced the incidence of chronic pain relative to thoracotomy, but it did not eliminate it: a recent systematic review reported that more than one-third of patients still experienced chronic postsurgical pain (CPSP) three months or more after VATS, with roughly one in ten reporting moderate-to-severe symptoms [1]. From the standpoint of the operating surgeon, this residual burden reframes postoperative pain not as a solved problem inherited from the thoracotomy era, but as an unfinished one that follows each successive generation of minimally invasive technique.

Robot-assisted thoracic surgery (RATS) was introduced into this landscape with considerable promise. Articulated wristed instruments, tremor filtration, and three-dimensional magnified visualization were expected to translate into gentler tissue handling and, by extension, less intercostal nerve trauma than rigid VATS instrumentation [2]. For many thoracic surgeons, this technical narrative carried an implicit corollary: greater precision should mean less pain. Subsequent comparative data have complicated that assumption. Randomized and propensity-matched studies directly comparing RATS with VATS have generally found comparable—rather than superior—acute and chronic pain outcomes [3,4], and several reports even suggest that multiport RATS may produce more early postoperative pain than uniportal VATS, presumably because port number and external arm mechanics outweigh any gain from intracorporeal articulation [5,6]. The gap between the theoretical appeal of robotic precision and its measured effect on pain is therefore not a minor footnote; it is a central, still-unresolved question for surgeons choosing among available minimally invasive platforms.

This question matters beyond patient comfort. Uncontrolled acute pain is a recognized driver of CPSP [1], and CPSP in turn compromises pulmonary rehabilitation, prolongs opioid exposure, and can delay or derail the delivery of adjuvant oncologic therapy. The concept of Return to Intended Oncologic Treatment (RIOT), first proposed by Aloia et al. as a quality metric for oncologic surgery, captures this downstream consequence directly: patients who cannot tolerate or timely initiate planned adjuvant therapy after surgery may forfeit part of the survival benefit that the operation was intended to provide [7]. In lung cancer specifically, delayed or incomplete adjuvant chemotherapy has been associated with worse survival [8], and enhanced recovery pathways that improve pain control have been shown to increase chemotherapy completion rates after resection [9]. Framed this way, postoperative pain management ceases to be solely a comfort-oriented, anesthesiology-driven endpoint and becomes a shared perioperative target linking surgical technique, anesthetic strategy, and oncologic outcome—precisely the intersection this Special Issue on Advanced Technologies in Thoracic Anesthesia is positioned to address. This narrative review accordingly examines pain outcomes across VATS and RATS from the operating surgeon’s perspective, the mechanisms and risk factors underlying their persistence, and the emerging role of RIOT as an integrative endpoint for evaluating minimally invasive thoracic surgery.

## 2. Literature Search

We conducted a non-systematic, targeted search of PubMed/MEDLINE from January 2010 through July 2026, reflecting the period since RATS entered routine thoracic oncologic practice. Search terms combined “robot-assisted thoracic surgery,” “RATS,” “video-assisted thoracoscopic surgery,” “VATS,” “pain,” “chronic postsurgical pain,” “intercostal nerve injury,” and “Return to Intended Oncologic Treatment” or “RIOT,” using Boolean operators. Randomized controlled trials, prospective and retrospective cohort studies, and systematic reviews/meta-analyses reporting acute or chronic pain outcomes after VATS or RATS lobectomy or segmentectomy were prioritized; case reports, conference abstracts, and non-English-language studies without an accessible translation were excluded. Reference lists of key trials and recent systematic reviews were hand-searched to identify additional relevant studies. The final search was completed in July 2026; the representative search string used for PubMed/MEDLINE is provided in Supplementary File S1. Titles and abstracts, followed by full texts, were screened by the corresponding author (T.M.) and a second author (R.D.), with disagreements resolved by discussion with the senior author (K.M.); where multiple publications reported overlapping cohorts, the report with the longest follow-up or most complete pain-outcome data was prioritized, and the others were cited only where they added information (e.g., long-term survival) not available in the primary report. A small number of relevant non-English-language studies and case reports (such as first-in-human reports of newly introduced robotic platforms (see Section 8)) were retained where they provided otherwise unavailable mechanistic or first-in-human data; these are identified as such in the text. As a narrative rather than systematic review, this search was not intended to be exhaustive or to follow PRISMA methodology; and no formal risk-of-bias or study-quality assessment was performed; rather, it aimed to identify the most methodologically robust and clinically representative evidence available to critically appraise the comparative pain outcomes and oncologic implications discussed in the sections that follow. This targeted approach carries an inherent risk of selection bias, which is acknowledged as a limitation in Section 10.

## 3. Definitions and Mechanisms

CPSP is formally defined, under the International Association for the Study of Pain (IASP) classification adopted for ICD-11, as pain that develops or intensifies after a surgical procedure, localizes to the surgical field, and persists for at least three months once the acute healing phase has resolved, provided that infection, malignant recurrence, or a pre-existing pain disorder can be reasonably excluded [10]. Within thoracic surgery, this entity is conventionally termed post-thoracotomy pain syndrome (PTPS) when it follows open resection, though the same diagnostic logic extends to VATS and RATS, where the qualifying descriptor becomes somewhat imprecise given the absence of a formal thoracotomy [11]. The three-month threshold is a pragmatic convention rather than a biological boundary; a substantial proportion of patients who eventually meet CPSP criteria already exhibit elevated pain scores in the first postoperative week, underscoring that chronicity is best understood as a continuum originating in the acute perioperative period rather than a discrete late event [12].

**Figure 1:**
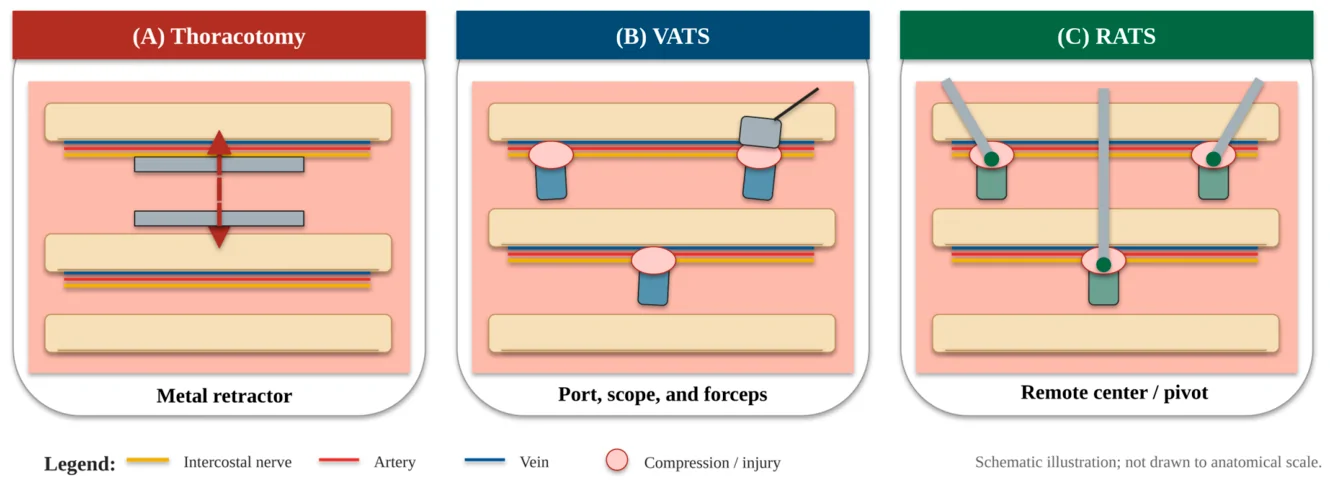
Mechanisms of Intercostal Nerve Injury by Surgical Approach.

From an anatomical standpoint, each intercostal space contains a neurovascular bundle running along the inferior costal groove, arranged from superior to inferior as vein, artery, and nerve. Any transthoracic access—regardless of platform—necessarily traverses this space, placing the nerve at risk of direct laceration, thermal injury from energy devices, or, more commonly, mechanical compression and traction at the point where an instrument, trocar, or retractor blade contacts the rib margins. This shared anatomical constraint is the reason no minimally invasive platform has proven inherently atraumatic to the intercostal nerve; each merely redistributes the mechanical insult differently. The specific loading pattern, however, does differ by approach (Figure 1).

Schematic illustration comparing the mechanisms of intercostal nerve injury during (A) thoracotomy, (B) video-assisted thoracoscopic surgery (VATS), and (C) robot-assisted thoracic surgery (RATS). In thoracotomy, rib spreading in the cranio-caudal direction produces sustained stretch and compression of the intercostal neurovascular bundle along the inferior rib margin. In VATS, rigid ports and thoracoscopic instruments may cause focal compression of the intercostal nerve at the port–rib interface. In RATS, the remote-center mechanism allows instruments to pivot around a fixed point at the chest wall; however, compression of the intercostal nerve may still occur at the port–rib interface. The intercostal neurovascular bundle is depicted within the costal groove along the inferior border of the rib. The illustration is schematic and not drawn to anatomical scale. The relative compression/injury markers shown are a conceptual illustration of the mechanistic hypothesis discussed in Section 3 and are not derived from a published biomechanical model or direct force measurement; they are qualitative rather than quantitative.

Posterolateral thoracotomy generates injury through forced rib spreading: a retractor widens a single intercostal space well beyond its physiological excursion, producing sustained stretch, localized ischemia, and, in a subset of cases, occult rib fracture [13]. Multiport VATS substitutes this wide single-incision trauma for narrower, focal loading at each trocar site, where the instrument pivots against the costal margins as though against a fulcrum during intracorporeal manipulation [14]. RATS was expected to relieve this fulcrum effect through wristed, tremor-filtered instrumentation, yet the rigid external arm remains fixed to the port and continues to transmit torque to the chest wall whenever the instrument tip executes wide-range motion; adjacent arms operating in proximity can additionally collide outside the thorax, adding an extracorporeal stress absent from VATS [5]. This mechanism appears to be accentuated in patients with a narrower intercostal space or shorter stature, in whom the same range of instrument motion imposes proportionally greater orthogonal load on the fixed port, a relationship demonstrated specifically for RATS but not reported for VATS [15] (OR 0.91 per 1 cm of height; detailed in Section 5). Multiport robotic configurations compound this effect further, since each additional arm adds another loaded fulcrum; several comparative studies accordingly report greater early postoperative pain with multiport RATS than with uniportal VATS despite the former’s superior intracorporeal precision [6,16].

Taken together, these observations support a mechanistic hypothesis—so far based on indirect clinical and port-configuration data rather than direct biomechanical measurement—that the theoretical advantages of robotic articulation operate predominantly on the intracorporeal side of the port, while extracorporeal transmission of force at the chest wall, the step most proximate to nerve injury, may remain comparatively unaddressed by current robotic design. Direct evidence quantifying port–rib force transmission for RATS versus VATS is currently lacking, and this mechanism should be regarded as plausible rather than demonstrated. If correct, this distinction would reframe the comparative pain data presented in the following section not as a paradox, but as a predictable consequence of where each platform’s engineering gains are concentrated.

## 4. VATS Versus RATS: Surgical Evidence on Pain Outcomes

Randomized evidence directly comparing pain after VATS and RATS remains sparse relative to the volume of retrospective series, but the trials that exist are notably consistent in what they do not show. The RVlob trial, the largest such study to date (*n* = 320), randomized patients to four-port robotic-assisted lobectomy or uniportal thoracoscopic lobectomy and found perioperative outcomes, visual analog pain scores, and complication rates to be statistically indistinguishable, despite the robotic arm harvesting significantly more lymph nodes [17]. A dedicated health-related quality-of-life substudy extended this comparison through 48 postoperative weeks and again found pain trajectories to be essentially superimposable between groups, with only a minor divergence at week four [3]; three-year survival data from the same trial have since reported non-inferior survival for the robotic arm [18]. It is worth noting that this flagship trial pitted a four-port robotic configuration against a single-port thoracoscopic one—an asymmetry that, per the mechanistic reasoning in Section 3, should have disadvantaged RATS—yet pain outcomes were merely equivalent rather than worse, illustrating that the port-number effect is not uniformly observed across all comparative designs.

Other randomized trials tell a similarly deflationary story. The ROMAN trial, an international multicenter effort, was terminated early at 83 patients when interim analysis showed a null probability that the robotic arm would outperform VATS on its primary composite outcome, though lymph node yield again favored RATS [2]. The Brazilian BRAVO trial found no difference in postoperative pain scores at day 3 or day 30 between platforms, with the only notable divergence being a lower 90-day readmission rate after RATS [19]. A smaller monocentric Italian trial did report a modest reduction in postoperative day-1 pain with RATS, alongside less pleural fluid accumulation, but did not extend follow-up into the chronic period [20]. The ongoing international RAVAL trial, designed explicitly to test the assumption—drawn largely from retrospective data—that RATS confers a genuine analgesic advantage, has so far reported comparable short-term health utility scores between arms in its early cost-effectiveness analysis, with definitive quality-of-life results still pending [21].

Non-randomized comparative cohorts reinforce this pattern across both acute and chronic timepoints. Kwon et al., testing the specific hypothesis that the robotic arm’s fulcrum rotation would spare the intercostal nerve relative to rigid VATS instrumentation, found no significant difference in either acute or chronic (Pain DETECT-assessed) pain between the two platforms [14]. Three-group comparisons incorporating open thoracotomy as a reference arm have similarly failed to separate RATS from VATS on postoperative pain or quality-of-life measures, even as robotic lymph node dissection outperformed both alternatives [22,23]. The one study extending direct comparison to a full year, evaluating VATS and RATS at two weeks, three months, six months, and twelve months, found numerically similar and gradually declining pain scores at every timepoint, with no evidence that either platform accelerated resolution of chronic pain [4]. A retrospective analysis using the EQ-5D-5L at six months to two years likewise found no significant difference in the proportion of patients reporting persistent pain, notwithstanding a numerically higher pain-free rate after VATS [24]. Perhaps most striking, one large-database analysis reported superior long-term quality-of-life metrics after VATS lobectomy compared with RATS—the inverse of the relationship these platforms were introduced to establish; as a large retrospective database analysis, however, this finding is susceptible to confounding by indication, surgeon experience, and unmeasured patient selection, and should be interpreted as hypothesis-generating rather than definitive [25].

A separate and clinically important line of evidence isolates port number as a confounder in its own right by comparing multiport RATS directly against uniportal VATS rather than against multiport VATS. Using this design, several groups have reported that multiport RATS produces greater pain during the first postoperative week—and, in one series, through postoperative day 14—than uniportal VATS, alongside greater early activity limitation, with differences narrowing or disappearing only after the first one to three weeks [5,16,26]. This conclusion is further reinforced by the largest prospective study to test it directly: a 12-institution Japanese cohort of 405 patients found that RATS could not demonstrate noninferiority to VATS on the primary pain endpoint at postoperative day 30 (82.0% vs. 94.7% with NRS ≤ 3), yet the RATS approach itself was not an independent risk factor for pain on multivariable analysis—only port number, wound size, tumor location, and use of epidural or continuous nerve block remained significant, and the gap was attributable in part to nearly half of the VATS cohort having undergone a uniportal procedure [27].

A direct comparison of multiport RATS against multiport VATS, controlling for port number on both sides of the comparison, has not, to our knowledge, been published; consequently, platform-specific factors such as external robotic-arm mechanics cannot be fully excluded as contributors to the pain differences described above, which are drawn entirely from studies contrasting multiport RATS with uniportal, not multiport, VATS. This body of evidence suggests that much of the pain differential attributed loosely to “RATS versus VATS” in the earlier literature may in fact reflect port number and configuration rather than the surgical platform itself, a distinction with direct implications for the uniportal robotic designs discussed in Section 8.

**Figure 2:**
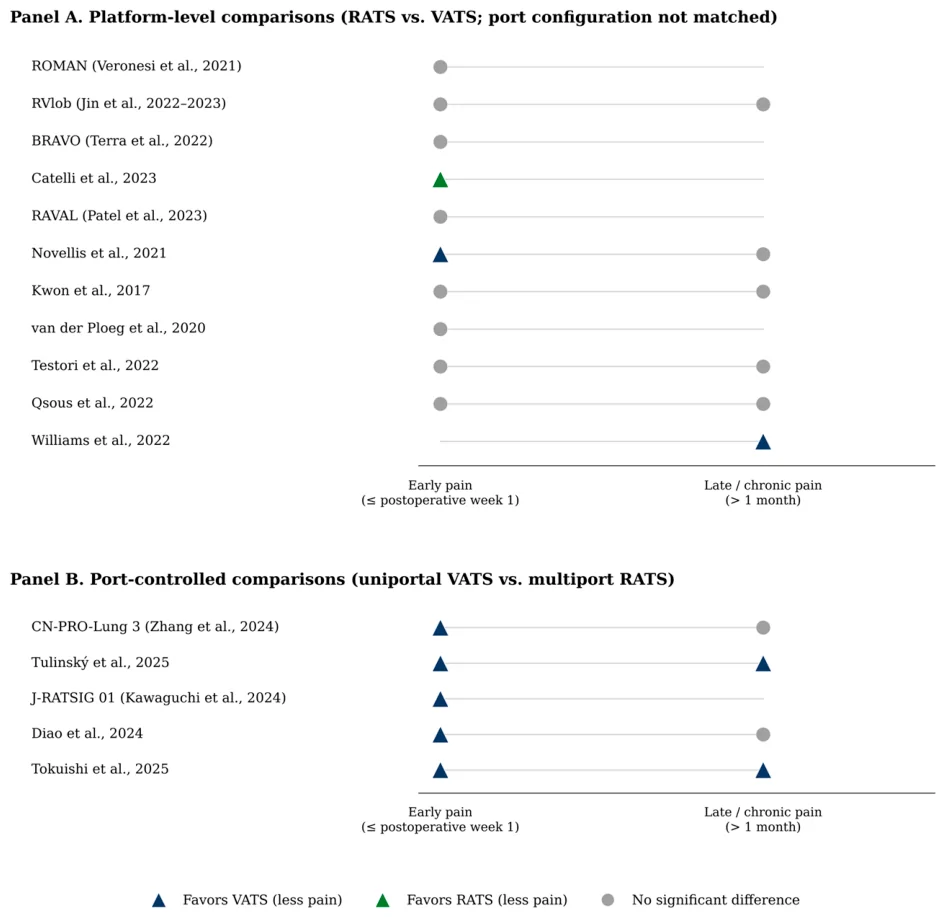
Direction of Effect for Postoperative Pain: Platform-Level versus Port-Controlled Comparisons [2,3,4,5,6,14,16,17,19,20,21,22,23,24,25,26,27].

A 2021 meta-analysis pooling over 11,000 patients confirmed that RATS offers measurable advantages in blood loss and conversion rate over VATS, but the same synthesis noted that pain outcomes were too heterogeneously reported across included studies to support pooled quantitative comparison [28]—an evidentiary gap that itself reflects how secondary a role pain has historically played in RATS-versus-VATS trial design. Considered together, the randomized, cohort, and port-controlled evidence converge on a single conclusion: robotic instrumentation has not delivered a consistent, clinically meaningful reduction in either acute or chronic pain relative to VATS, and where a difference does emerge, it favors VATS as often as it does RATS. The principal studies comparing pain outcomes between VATS and RATS are summarized in Table 1. To make this platform-versus-port distinction visually explicit, Figure 2 separates the direction of effect (favors VATS, favors RATS, or no significant difference) for platform-level comparisons (Panel A) from port-controlled comparisons (Panel B) at early and late/chronic timepoints; this is a qualitative summary of effect direction rather than a pooled quantitative meta-analysis, given the pain-instrument heterogeneity discussed in Section 10.

For each study in Table 1, markers indicate the direction of the reported pain difference at an early (≤postoperative week 1) and, where assessed, a late/chronic (>1 month) timepoint: filled triangles denote a statistically significant difference favoring VATS (dark blue) or RATS (green), and gray circles denote no statistically significant difference. Panel A includes platform-level comparisons in which port number was not matched between arms; Panel B restricts the comparison to studies that specifically contrasted uniportal VATS with multiport RATS, isolating the port-configuration effect discussed in Section 4. This figure summarizes effect direction only—not pooled effect size—because of the heterogeneous pain instruments and follow-up windows across studies; it should not be interpreted as a quantitative meta-analysis.

Procedure type and perioperative analgesic protocol are additional potential confounders of the comparisons in Table 1. Most of the platform-level trials (ROMAN [2], RVlob [3,17,18], BRAVO [19], Catelli et al. [20], RAVAL [21], Novellis et al. [22], van der Ploeg et al. [23]) and the port-controlled studies of Williams et al. [25], Tulinsky et al. [5], and Diao et al. [16] enrolled patients undergoing lobectomy; Kwon et al. [14], Tokuishi et al. [6], J-RATSIG 01 [27], and the Ma et al. [28] meta-analysis included a mixed population of lobectomy and segmentectomy.

Regional-analgesia protocols were reported with sufficient detail to summarize in only a minority of these studies: Kwon et al. [14] applied an identical protocol of port-site local anesthetic infiltration with intercostal nerve block and intravenous patient-controlled analgesia (PCA) to both the VATS and RATS arms; RAVAL specifies intravenous PCA managed by the hospital pain service, with no protocolized regional block; and Catelli et al. [20] state that “the same anesthetic protocol” was used in both arms without further detail. Notably, in J-RATSIG 01, use of epidural anesthesia or continuous nerve block differed significantly between arms (45% RATS vs. 31% VATS), which the original investigators themselves identified as a potential confounder of the observed pain difference—directly illustrating the analgesic-protocol confounding this section addresses. The remaining studies in Table 1 did not report their regional-analgesia protocol with enough detail in the publicly available abstract or methods for us to reliably summarize here.

## 5. Risk Factors for Chronic Pain: A Surgical Checklist

Patient-related, surgical, and postoperative risk factors associated with CPSP after minimally invasive thoracic surgery are summarized in Table 2.

Population-level predictors of CPSP—female sex, younger age, and preoperative anxiety—are consistently identified across meta-analytic syntheses but lie outside the surgeon’s control [1,29]. From an operative standpoint, the more actionable risk factors are those embedded in technical decision-making, and here the literature is more specific than is often appreciated. A pooled analysis spanning multiple cohorts identified port number as the single strongest surgically modifiable predictor of chronic pain, with three-port configurations carrying more than four times the odds of CPSP relative to fewer-port approaches, alongside smaller but significant contributions from prolonged operative time and extended chest-tube dwell time [1,30]. Each of these factors converges mechanistically on the same pathway described in Section 3: more ports mean more loaded fulcrums, longer operative time means more sustained instrument-transmitted force, and prolonged drainage means persistent mechanical irritation at the tube-bearing intercostal space.

Two further intraoperative variables warrant explicit attention because they represent genuine points of surgeon-level intervention rather than fixed patient characteristics. Formal intercostal suture closure at the port site has been identified as an independent protective factor, reducing the odds of chronic pain by roughly three-quarters relative to unsutured closure, while each additional centimeter of incision or port length independently increases risk [31]. Neither maneuver requires new technology or added operative time of consequence, making them among the few evidence-based, immediately implementable technical modifications available to the operating surgeon across any minimally invasive platform.

A risk factor specific to robotic surgery, and largely absent from the VATS literature, is patient stature. Multivariate analysis has identified shorter height—and, by extension, a narrower intercostal space—as an independent predictor of post-thoracotomy pain following RATS, with reasonable discriminative accuracy on receiver-operating-characteristic analysis [15]. This finding gives anatomical specificity to the orthogonal-loading mechanism proposed in Section 3: in patients with less intercostal room to accommodate the fixed external arm, a given range of instrument motion transmits proportionally greater force to the chest wall. It suggests that patient selection or port-site planning tailored to body habitus may be an underexplored lever for reducing RATS-specific pain.

Postoperatively, inadequately controlled acute pain remains one of the most consistently reported predictors of chronicity across surgical platforms, reinforcing that intraoperative technique and postoperative analgesic strategy are not independent variables but sequential links in the same causal chain [1,11]. A validated early screening measure—pain on shoulder abduction during the first six postoperative days—has shown the strongest discriminative performance of any bedside assessment for identifying patients likely to develop chronic pain, offering a practical, low-cost tool for early risk stratification that surgical teams can apply before formal chronic-pain criteria are even met [31].

## 6. Postoperative Pain as a Barrier to Functional Recovery and RIOT

The clinical significance of postoperative pain extends well beyond patient comfort. Poorly controlled pain restricts chest wall excursion, discourages effective coughing and deep breathing, and delays ambulation—each of which raises the risk of atelectasis and pneumonia and, in turn, prolongs chest-tube dwell time and hospital length of stay. Because both of these variables are themselves independent predictors of chronic pain (Section 5), inadequate acute analgesia initiates a self-reinforcing cycle in which pain begets immobility, immobility begets complications, and complications beget more pain. Opioid-based rescue analgesia, the default response to this cycle in many units, compounds the problem through sedation, ileus, and further deconditioning, so that the net effect of undertreated pain is a measurably slower trajectory back to a patient’s functional baseline rather than a discomfort confined to the immediate postoperative days.

This functional deficit has direct oncologic consequences, formalized in the concept of Return to Intended Oncologic Treatment (RIOT). First proposed by Aloia et al. as a quality metric for cancer surgery broadly, RIOT quantifies the proportion of patients who are able to receive their planned adjuvant therapy—and the interval before they do so—reasoning that a technically successful operation offers only partial value if the patient’s postoperative course prevents timely delivery of the systemic treatment on which long-term oncologic control depends [7]. Applied specifically to lung cancer, this framing has particular force: Donington and Martin, commenting on the broader relevance of RIOT to thoracic oncology, argue that surgeons should evaluate perioperative pathways not solely by 30- or 90-day morbidity but by whether patients reach the medical oncologist’s office intact and on schedule [32].

As Table 3 makes explicit, none of the studies underlying the RIOT concept in this literature directly measured postoperative pain and an oncologic-treatment endpoint within the same analysis. The pain–RIOT link discussed in this section is therefore an inference constructed by juxtaposing two separate bodies of evidence—the pain literature reviewed in Section 4 and Section 5 and the RIOT/adjuvant-therapy literature summarized here—rather than a relationship any single study has empirically demonstrated. This evidentiary gap is precisely why we describe the link as a plausible, testable hypothesis rather than an established causal pathway (see Section 10).

The stakes of this metric are not abstract, although the relationship with timing is more forgiving than commonly assumed: a large National Cancer Database cohort found that adjuvant chemotherapy initiated up to 127 days (roughly four months) after resection was not associated with increased mortality compared with earlier initiation, and patients treated within this extended window still had substantially lower mortality than those who received surgery alone [8]. Based on this single retrospective cohort, the more consequential factor, in other words, appears to be not a specific number of days but whether the patient ultimately receives treatment at all—precisely the outcome that uncontrolled pain and its downstream complications (Section 5) put at risk.

Enhanced recovery after surgery (ERAS) pathways, in which multimodal analgesia and opioid-sparing regional techniques are central components rather than adjuncts, provide the clearest empirical bridge between perioperative pain management and RIOT in thoracic surgery specifically. A large single-institution study found that adjuvant chemotherapy completion rose from approximately 40% in the pre-ERAS era to 62% after full ERAS implementation, with a significantly shortened interval to treatment initiation; notably, surgical approach itself—open versus thoracoscopic—was not independently associated with completing adjuvant therapy once ERAS status was accounted for [33]. This finding is instructive for the present review: it suggests that the perioperative care pathway, of which pain control is a principal driver, may matter more for RIOT than the choice of minimally invasive platform per se. Complementary work has framed postoperative complications generally as the mechanistic link between surgical care and RIOT, positioning anesthesiologic and surgical teams as joint custodians of a single perioperative outcome rather than as sequential, independently accountable stages of care [34].

This reframing carries a direct implication for the VATS-versus-RATS debate examined in Section 4. If platform choice alone were sufficient to improve RIOT, one would expect RATS—marketed in part on the promise of gentler recovery—to outperform even open thoracotomy on chemotherapy completion. Yet a retrospective comparison of adjuvant chemotherapy completion after RATS versus thoracotomy found no significant difference between the two (74.3% vs. 75.8%), despite RATS patients having smaller, lower-stage tumors that might otherwise have favored earlier treatment [35]. Taken together with the pain data in Section 4, this result reinforces a central argument of this review: surgical platform selection is not, by itself, an effective lever for improving oncologic recovery, and the more consequential target is the quality of perioperative pain and complication management layered on top of whichever platform is used. For a Special Issue devoted to advanced technologies in thoracic anesthesia, this positions pain control not as a downstream comfort measure but as an upstream determinant of whether the intended oncologic treatment plan is actually delivered.

## 7. Multimodal Analgesia: A Brief Overview from the Surgical Standpoint

Multimodal, opioid-sparing analgesia is now the standard of care for minimally invasive thoracic surgery [36], formalized in both the ERAS Society/European Society of Thoracic Surgeons guidelines and procedure-specific PROSPECT recommendations for VATS [37,38]. These guidelines converge on a common architecture: regular acetaminophen and NSAIDs as a baseline, a form of regional anesthesia—paravertebral, erector spinae plane, or serratus anterior plane blockade—as the primary opioid-sparing component, and opioids reserved as rescue rather than first-line therapy [38]. Within this regional-anesthesia component, current evidence and consensus guidelines favor paravertebral and erector spinae plane blocks as first-line techniques for VATS and RATS, with thoracic epidural analgesia now reserved chiefly for open thoracotomy or for cases converted from a minimally invasive approach [37,38].

The acetaminophen/NSAID baseline itself requires individualized judgment rather than routine, unselected use: NSAIDs should be used cautiously, or avoided, in patients with renal impairment. In selected patients, particularly those with a neuropathic pain component, gabapentinoids may also have a role, though the supporting evidence is mixed and the adverse-effect profile—sedation, dizziness, and a recognized dependence liability—argues against routine, unselected use. Clinical studies, including our previous work, have evaluated gabapentinoids in patients with post-thoracotomy neuropathic pain [39,40].

The comparative merits of these anesthesiologist-administered regional techniques are addressed in depth elsewhere in this Special Issue; from the surgeon’s chair, the more immediately relevant question is which components of multimodal analgesia lie within the surgical field itself and under direct surgical control. Two such techniques warrant specific comment. The first is intraoperative intercostal nerve blockade under direct thoracoscopic or robotic vision, typically performed with long-acting local anesthetic at the conclusion of the case; together with the intercostal suture technique discussed in Section 5, this represents one of the few analgesic interventions a surgeon can deliver without additional equipment, specialist input, or meaningful added operative time. Despite requiring no additional equipment, adoption of this technique varies considerably across institutions and surgeons, reflecting differences in training, unfamiliarity with optimal technique and timing, and the absence of a standardized institutional protocol in many centers; addressing these modifiable barriers, rather than the technique’s intrinsic effectiveness, may be the more immediate opportunity for wider uptake.

The second is intraoperative intercostal nerve cryoablation, in which a cryoprobe freezes the nerve under direct visualization to produce temporary conduction block. Early retrospective series reported increased short-term opioid requirements after cryoablation in RATS cohorts [41], but this promise has not held up under randomized scrutiny: a recent randomized trial found no meaningful reduction in opioid consumption or in-hospital pain scores with cryoablation added to standard intercostal blockade, and instead reported increased neuropathic pain at two weeks in the cryoablation group [42]. This divergence between early observational enthusiasm and subsequent randomized disappointment closely parallels the broader pattern this review has traced for RATS itself, and is a useful reminder that surgically appealing, technologically mediated interventions warrant the same rigor of comparative evaluation as the surgical platforms that host them.

None of these surgeon-administered adjuncts substitutes for the structural, technique-level factors identified in Section 5. A well-executed nerve block cannot fully offset the added risk conferred by an unnecessary fourth port, nor can cryoablation compensate for prolonged operative time or delayed drain removal. Multimodal analgesia and surgical technique are therefore best understood as complementary rather than interchangeable levers, both of which report to the same downstream target established in Section 6: getting the patient through the perioperative period with enough functional reserve, and little enough residual pain, to proceed to intended oncologic treatment on schedule.

## 8. Emerging Robotic Platforms: Surgical Perspective on Pain-Relevant Design

If port number is, as Section 3 and Section 5 have argued, the single most consistently identified surgically modifiable driver of chronic pain, then the most logical next step in robotic platform design is not incremental refinement of multi-arm articulation but outright reduction of the number of fulcrums placed on the chest wall, or a redesign of how force is transmitted at each fulcrum. The early data from several next-generation platforms—both single-port and Japan-developed multiport systems—offer a useful test of whether this mechanistic reasoning translates into measurable benefit.

The da Vinci SP (single-port) system (Intuitive Surgical, Sunnyvale, CA, USA) delivers a fully wristed camera and three articulated instruments through one 2.5–3 cm incision, eliminating the multi-port fulcrum arrangement described in Section 3. Early thoracic series have been reassuring on feasibility, with modest pain scores and short chest tube duration reported across small case series [43,44]. The most informative data to date come from a 2026 propensity-score-matched comparison of single-port RATS, multiport RATS, and VATS segmentectomy: single-port RATS achieved significantly shorter chest tube duration than both comparators, yet postoperative pain did not differ significantly across any of the three groups [45]—a finding that again separates mechanistic plausibility from measured outcome. Subxiphoid and subcostal single-port approaches to anterior mediastinal disease extend this logic furthest, avoiding the intercostal space altogether by routing the port beneath the sternum rather than between ribs [46].

Two domestically developed Japanese platforms illustrate an alternative design philosophy. The hinotori surgical robot system (Medicaroid Corporation, Kobe, Japan), approved for reimbursed thoracic use in Japan since June 2024, uses a software-controlled pivot point that eliminates mechanical docking between the robotic arm and the port—in principle reducing the rigid, fixed-fulcrum force transmission characteristic of docked multiport RATS described in Section 3. Early comparative data report perioperative outcomes broadly comparable to the da Vinci platform in primary lung cancer surgery [47], and a small trocarless series using wound retractors instead of rigid ports has further reduced port-site trauma at the docking interface, though without dedicated pain endpoints [48]. Saroa (Riverfield Inc., Tokyo, Japan), a pneumatically driven system providing real-time haptic force feedback, likewise requires no port docking and has been used clinically for lobectomy and segmentectomy, with force-feedback data suggesting gentler intraoperative tissue handling than conventional robotic grasping [49]. Neither platform has yet reported a dedicated, adequately powered comparison of postoperative pain against VATS or da Vinci RATS, and both remain, for now, feasibility- and non-inferiority-stage evidence rather than confirmed analgesic advances.

This gap is instructive rather than merely a limitation to note in passing. Given how consistently this review has shown that theoretical mechanical advantages fail to predict measured pain outcomes—from RATS itself in Section 4 to single-port platforms above—extrapolating a pain benefit from docking-free pivot design or haptic feedback without dedicated comparative data would repeat the same reasoning error that shaped early expectations of multiport RATS. Future evaluations of these platforms, Japanese-developed systems included, would be better served by incorporating pain and RIOT as prespecified outcomes from their first comparative series, rather than appending them retrospectively once feasibility has already been established.

## 9. Future Directions: Integrating Surgical and Anesthetic Strategies

The heterogeneity evident throughout Table 1—differing pain instruments, inconsistent follow-up windows, and pain relegated to a secondary or exploratory endpoint in most trials—is itself a barrier to progress. Adoption of a core outcome set modeled on the IMMPACT framework, which specifies pain intensity, physical functioning, and rescue analgesic use as minimum reportable domains, alongside movement-evoked rather than only resting pain scores, would allow future VATS-versus-RATS comparisons to be pooled meaningfully rather than narratively summarized as they have been here [50,51]. Without this standardization, each new robotic platform risks generating another isolated feasibility series rather than evidence that accumulates. Concretely, we propose that future comparative trials assess this core outcome set at a minimum of five timepoints—postoperative days 1–3, 30 days, and 3, 6, and 12 months—with total opioid consumption (in oral morphine equivalents) and rescue-analgesic requirement reported alongside pain intensity at each timepoint, rather than pain alone.

RIOT offers a natural extension of this same standardization effort. Rather than being assessed retrospectively, as in the studies discussed in Section 6, RIOT and its component metrics—time to adjuvant therapy initiation, completion rate, and dose intensity, together with a functional-recovery milestone such as time to return to baseline activities of daily living—should be prespecified secondary endpoints in trials of new surgical platforms from their first comparative series onward, including the single-port and Japanese-developed systems discussed in Section 8. The ongoing RAVAL trial, with its multi-year follow-up incorporating patient-reported outcomes, offers one template for this design, though it does not yet capture RIOT explicitly; future protocols would benefit from building oncologic treatment delivery into their outcome hierarchy alongside pain and quality of life from the outset.

Most fundamentally, the surgical and anesthetic studies on thoracic pain have developed largely in parallel, with surgical trials reporting platform and technique while anesthetic trials report block type and analgesic regimen, rarely as covariates in the same model. Given that this review has shown surgical factors—port number, operative time, intercostal suture use—to carry effect sizes comparable to or larger than those typically reported for individual regional anesthesia techniques, future trial designs should treat surgical technique and anesthetic strategy as interacting variables within a single factorial or registry-based framework rather than as separately reported studies. A Special Issue bringing together advanced anesthetic technology with thoracic surgical practice is well-positioned to model this integration directly, both by pairing surgical and anesthetic contributions on shared patient cohorts and by encouraging joint authorship between the two specialties on future comparative trials. Only through this combined lens is the field likely to determine which interventions—surgical, anesthetic, or their combination—meaningfully shift the pain and RIOT outcomes this review has shown neither specialty has yet solved alone.

## 10. Limitations

Several limitations should be considered when interpreting this review. First, as a targeted narrative rather than a systematic review, study selection was not exhaustive, was not guided by a PRISMA-type protocol, and did not include formal risk-of-bias or study-quality assessment; the search strategy is summarized in Section 2 and provided in Supplementary File S1, but selection bias favoring studies the authors judged clinically representative cannot be excluded. Second, much of the comparative evidence originates from high-volume, often geographically concentrated centers, where surgeon experience, institutional learning curves, ERAS implementation, and regional-anesthesia practice may not generalize to lower-volume or differently resourced settings, limiting external validity. Third, the included studies use heterogeneous pain instruments (NRS, VAS, painDETECT, EQ-5D-5L, and disease-specific quality-of-life measures) and inconsistent follow-up windows, and these acute, chronic, and neuropathic pain endpoints, along with functional interference and quality of life, are not interchangeable outcomes; this heterogeneity, summarized in Table 1 and Table 2, precludes pooled quantitative synthesis and is a principal motivation for the core-outcome-set proposal in this section. Fourth, several comparisons that nominally test “RATS versus VATS” in fact confound platform with port number and access configuration, and the associations reported in Table 2, particularly those derived from observational and propensity-matched rather than randomized designs, remain susceptible to residual confounding by procedure complexity, patient anatomy, and surgeon preference; these associations should not be read as demonstrated causal effects. Finally, the relationship between pain, functional recovery, and RIOT discussed in Section 6 is supported by indirect and largely observational evidence; a direct, independent causal effect of postoperative pain on RIOT or long-term survival has not been established and remains an important target for prospective study.

## 11. Conclusions

Minimally invasive thoracic surgery has substantially reduced the pain burden associated with lung resection relative to open thoracotomy, but the expectation that robotic assistance would meaningfully extend this improvement over VATS has not been borne out by the accumulated randomized, cohort, and propensity-matched evidence reviewed here. Acute and chronic pain outcomes after RATS are, at best, comparable to VATS, and in several port-controlled comparisons, multiport robotic platforms have produced more pain than uniportal thoracoscopic approaches—an inversion of the relationship these platforms were introduced to establish. This pattern is not paradoxical once viewed mechanistically: the intercostal neurovascular bundle is loaded at the port margin regardless of platform, and robotic articulation improves precision almost entirely on the intracorporeal side of that margin, leaving the extracorporeal transmission of force at the fixed port largely unaddressed by current design.

From a surgical standpoint, this reframes pain not as an inherent, unmodifiable cost of minimally invasive access, but as a variable substantially within the operating surgeon’s control—through port number, operative time, drainage duration, and simple technical measures such as intercostal suture closure—independent of which platform is chosen. It also reframes pain itself: not merely a comfort endpoint but, through its documented effect on functional recovery and Return to Intended Oncologic Treatment, a plausible determinant of the oncologic benefit surgery is meant to confer. Emerging single-port and Japanese-developed robotic platforms offer mechanistically plausible routes toward reducing this pain burden further, but, following the same pattern seen with first-generation RATS, their promise should be regarded as a hypothesis to be tested rather than a benefit to be assumed. Realizing genuine progress will require the surgical and anesthetic communities to jointly treat pain control, RIOT, and long-term survival as linked, prespecified outcomes in the trials that evaluate the next generation of thoracic surgical technology.

## Figures and Tables

**Table 1:** VATS versus RATS: pain outcomes across randomized, cohort, and port-controlled studies.

Category	Study/Trial	Design and Sample Size	Assessment Timepoint(s)	Pain Assessment Tool	Key Findings Related to Pain	Reference(s)
A. Multicenter RCT	ROMAN trial (international)	*n* = 77 (VATS 39/RATS 38)	Daily postoperative assessment	Daily pain score EORTC QLQ-C30	No significant difference in the primary composite outcome. Data on QoL, pain, and recurrence require a longer follow-up time to have a complete recording and are not reported in this initial analysis.	[2]
	RAVAL trial (international)	Phase A: *n* = 164 (VATS 83/RATS 81)	POD1 weeks 3, 7, 12 months 6, 12	EQ-5D-5L	The mean 12-week health utility score was significantly higher with RATS (*p* = 0.02), although overall short-term health utility was broadly comparable between groups.	[21]
B. Single-center RCT	RVlob trial (China)	*n* = 320 (VATS 163/RATS 157)	POD1; weeks 4, 24, 48; 3 y	NRS EORTC QLQ C30 EQ-5D	Perioperative outcomes were comparable. Pain and HRQoL were similar between groups through week 48, with only a minor difference at week 4. Three-year survival was non-inferior (RATS 94.6% vs. VATS 91.5%).	[3,17,18]
	BRAVO trial (Brazil)	*n* = 76 (VATS 39/RATS 37)	POD3, 30	Postoperative pain score defined as a score >2 in VAS	No significant difference in the primary 90-day mortality endpoint. No significant difference in pain at day 3 or day 30.	[19]
	Catelli et al., 2023 (Italy)	*n* = 75 (VATS 50/RATS 25) 1:2 allocation	POD1	VAS	VAS on postoperative day 1 was significantly lower with RATS (0.92 vs. 1.17, *p* = 0.005). Long-term pain was not assessed.	[20]
C. Multicenter Prospective Cohort Studies	J-RATSIG 01 (Japan)	*n* = 405 (VATS 209/RATS 196) half of VATS; uniportal	POD 10, 30, 90	NRS painDETECT	Primary endpoint (NRS ≤ 3 at POD30): 82.0% (RATS) vs. 94.7% (VATS); noninferiority not demonstrated (95%CI −19.0 to −6.6)	[27]
D. Single-center Prospective Cohort Studies	Novellis et al., 2021	*n* = 169 (Open 58/VATS 58/RATS 53)	POD3; weeks 2; months 6,12	NRS Brief Pain Inventory EORTC QLQ-C30 QLQ-LC13	In the short term, VATS is associated with less pain than RATS, whereas in the long term, there is virtually no difference in pain intensity between the two procedures. Additionally, no significant difference was observed in the rate of analgesic use.	[22]
E. Single-center Retrospective Cohort Studies	Kwon et al., 2017	*n* = 502 (Open 201/VATS 227/RATS 74)	POD 1–9; chronic follow-up	NRS painDETECT	No significant difference in either acute or chronic pain between RATS and VATS. The hypothesis that fulcrum rotation of the robotic arm spares the intercostal nerve was not supported.	[14]
	van der Ploeg et al., 2020	*n* = 57 (Open 18/VATS 17/RATS 22)	During hospitalization	NRS	No significant differences by surgical approach, age, sex, right-sided surgery, postoperative NRS score, drain duration, epidural analgesia duration, or length of stay.	[23]
	Testori et al., 2022	*n* = 100 (VATS 50/RATS 50)	2 weeks; 3 months, 6 months; 1 year	NRS	Median NRS (VATS/RATS) at each timepoint: 2 weeks 2.96/2.86; 3 months 2.16/2.06; 6 months 1.62/1.56; 1 year 1.30/1.24. No significant difference at any timepoint.	[4]
	Qsous et al., 2022	*n* = 92 (VATS 51/RATS 41)	6 months to 2 years	EQ-5D-5L	Pain-free rate at assessment: 62.7% (VATS) vs. 51.2% (RATS); mild pain: 25.5% (VATS) vs. 39% (RATS) No significant acute or long-term pain advantage of RATS over VATS was demonstrated.	[24]
	Williams et al., 2022	*n* = 219 (VATS 139/RATS 80)	1, 6, and 12 months	QLQ-C30 QLQ-LC13	VATS demonstrated significantly better postoperative outcomes (*p* < 0.05), including higher QLQ-C30 summary, social, and cognitive score. At 6 months postoperatively, VATS patients reported significantly lower QLQ-LC13 symptom burden—specifically reduced dyspnea, neuropathy, and pain	[25]
F. Prospective cohort Studies Addressing the Port-Number Confounder	CN-PRO- Lung 3	Propensity-score matched (Uniportal VATS 148/, Multiportal RATS 148)	During hospitalization to 4 weeks	Perioperative Symptom Assessment for Lung Surgery	In-hospital pain severity (*p* = 0.019) and activity limitation (*p* = 0.001) were significantly greater in the multiport RATS group, but no significant differences remained after discharge, including pain. A counterintuitive finding that multiport RATS may cause more acute pain than uniportal VATS.	[26]
	Tulinský et al., 2025	*n* = 140 (Uniportal VATS 70/ RATS 70)	POD3 and 14	VAS DAPIQ (pain interference with daily activities)	VAS was significantly higher with RATS than Uniportal VATS at both POD3 (5.8 vs. 3.8, *p* < 0.001) and POD14 (2.7 vs. 2.2, *p* = 0.001). Pain-free rate at POD14: RATS 17.1% vs. Uniportal VATS 34.3% (*p* = 0.016). RATS carried a 3.8-fold higher risk of pain-related activity limitation.	[5]
G. Retrospective cohort Studies Addressing the Port-Number Confounder	Diao et al., 2024	Propensity-score matched (Uniportal VATS 42/3-port RATS 42)	1–3 week	NRS Christensen Fatigue Scale EORTC QLQ-C30	Pain severity was greater with RATS than with uniportal VATS through postoperative week 1, with no difference at weeks 2–3. RATS carries greater short-term pain but fewer complications.	[16]
	Tokuishi et al., 2025	Propensity-score matched (Uniportal VATS 52/4-port RATS 52)	POD 1–7 analgesic use over POD 60	NRS	Uniportal VATS pain scores were significantly lower than RATS throughout the postoperative course and lower analgesic use	[6]
H. Systematic Reviews and Meta-Analyses	Ma et al., 2021	18 studies, *n* = 11,247 (VATS 6133/RATS 5114)	Perioperative	not included	Pooled quantitative analysis of pain was insufficient—an area requiring further meta-analytic synthesis.	[28]

RCT: Randomized controlled trial; EORTC QLQ-C30: European Organisation for Research and Treatment of Cancer Quality of Life Questionnaire—Core 30; EORTC QLQ-LC13: European Organisation for Research and Treatment of Cancer Quality of Life Questionnaire—Lung Cancer 13; EQ-5D-5L: EuroQol 5-Dimension 5-Level instrument; VAS: Visual analogue scale; NRS: Numeric rating scale; POD: Postoperative day; VATS: Video-assisted thoracoscopic surgery; RATS: Robotic-assisted thoracoscopic surgery.

**Table 2:** Risk factors for chronic postsurgical pain after minimally invasive thoracic surgery.

Category	Risk Factor	Odds Ratio/Statistics	Proposed Mechanism	Surgical Relevance/Clinical Application	Reference(s)
A. Patient- Related Factors	Female sex	OR 1.58 (95% CI 1.20–1.96)	Sex-related differences in pain threshold/tolerance and neuroimmune mechanisms are thought to contribute	Not modifiable by surgical technique. Relevant for identifying high-risk patients for perioperative counseling	[1]
	Younger age	OR 0.92 (older age protective) (95% CI 0.85–0.99)	Age-related increase in pain threshold; differences in pain-reporting behavior	Not modifiable by surgical technique. Acute pain management should be intensified in younger patients	[1]
	Preoperative anxiety	OR 3.09 (95% CI 1.20–7.94)	Promotes central sensitization and pain catastrophizing	Adequate preoperative counseling and psychological preparation are an important part of perioperative management	[29]
	Pre-existing pain	OR 2.56 (95% CI 1.70–3.88)	Pre-existing sensitization may amplify postoperative central sensitization	Should be screened for during preoperative evaluation	[30]
	Short stature/narrow thoracic width (RATS-specific)	OR 0.91 per 1 cm in height (95% CI 0.84–0.98)	In patients with a narrow intercostal space, robotic port insertion imposes greater chest-wall load, and orthogonal instrument movement more readily compresses the intercostal nerve	Particular attention to port placement and trocar selection is warranted in shorter patients undergoing RATS. An important RATS-specific anatomical risk factor	[15]
B. Surgical/ Intraoperative Factors	Port number (3 ports vs. fewer ports)	OR 4.32 (95% CI 3.51–5.13)	Increasing port number directly increases the number of sites at risk for intercostal nerve injury	Observational evidence, potentially confounded by procedure complexity and surgeon experience, supporting further evaluation of uniportal or port-reduction strategies; suggests but does not prove that multiport RATS configurations increase CPSP risk	[1]
	Prolonged operative time	OR 1.007 per minute (95% CI 1.00–10.01)	Prolonged port/instrument dwell time increases intercostal nerve compression and sustained tissue traction	Risk may be higher during the early RATS learning curve, when operative times tend to be longer; improved procedural efficiency may help prevent pain	[1]
	Prolonged chest-tube dwell time	OR 1.08 (95% CI 1.05–1.12)	Sustained compression and irritation of the intercostal nerve at the drain insertion site	Early drain-removal protocols (as part of ERAS) may help prevent pain	[1]
	Tumor location (upper vs. lower lobe)	OR 2.416 (95% CI 1.06–5.51)	Upper-lobe resections generally require a larger trocar angle, which may increase intercostal nerve strain	Consider tailoring port placement/angle for upper-lobe procedures specifically	[27]
	Use of intercostal suture closure	OR 0.23 (95% CI 0.07–0.74)	Proper suture closure at the port site may reduce sustained compression and inflammation of the intercostal nerve	Observational evidence suggesting a possible protective effect and within surgeons’ direct control; a candidate technique for prospective evaluation and procedural standardization	[31]
	Incision/port length	OR 1.240 per 1 cm (95% CI 1.05–1.47)	Longer incisions correlate with a greater area of tissue injury and a wider zone of intercostal nerve involvement	Minimizing port/incision length where feasible may help prevent pain	[31]
	Surgical approach (RATS vs. VATS; multiport vs. uniportal)	multiport RATS is associated with more acute pain than uniportal VATS in several comparative studies (see Table 1)	Increased port number plus additional chest-wall stress from external collision of robotic arms	Evidence warranting re-examination of the assumption that “RATS = less pain”; supports the rationale for emerging technologies such as uniportal RATS	[5,6,16,26,27]
C. Postoperative Factors	Acute postsurgical pain (APSP)	OR 1.84 (95% CI 1.57–2.12)	Strong nociceptive input during the acute phase induces central sensitization and synaptic plasticity changes that progress to chronicity	Rigorous acute pain control is the single most important target for CPSP prevention, and a perioperative management issue directly linked to the RIOT concept	[1,11]
	Insufficient postoperative analgesia	OR 0.54 Adequate analgesia; protective (95% CI 0.17–0.91)	Preventing prolongation of acute pain suppresses progression of central sensitization	Early implementation of multimodal analgesia and regional anesthesia is important (detailed in Section 7)	[1]
	Pain on shoulder abduction (POD 1–6)	OR 1.89 (95% CI 1.43–2.50)	A composite pain measure reflecting involvement of muscles and nerves of the shoulder girdle	Clinically useful as an early postoperative screening tool; applicable for early identification of high-risk patients	[31]
	Adjuvant chemotherapy	OR 1.58 (95% CI 1.07–2.09)	Chemotherapy-induced tissue edema may worsen pre-existing nerve injury via a “double crush”–like mechanism and promote central sensitization	Active perioperative pain management and monitoring is recommended in patients eligible for chemotherapy	[1]
	Wound infection/ complications	Reported as an independent predictor alongside rescue analgesic and sedative use	Prolonged inflammation sustains peripheral sensitization	Complication prevention (aseptic technique, appropriate wound care) also contributes to CPSP prevention	[30]
D. Socioeconomic and Other Factors	Education level (less than junior high school)	OR 1.27 (95% CI 1.05–1.48)	Lower educational attainment is associated with pain catastrophizing	Consider additional patient education and perioperative support for this psychosocially high-risk group	[1]
	Smoking/alcohol history	Smoking: OR 0.80 (95% CI 0.65–0.95) Alcohol: OR 0.77 (95% CI 0.60–0.94)	Possible confounding with sex; true biological protective effect is unclear	Other epidemiological studies report that smoking may instead increase pain sensitivity, so cautious interpretation is warranted	[1]

Notes on Table 2: source population, analysis type, and outcome definition, by reference. [1] Chen et al., Pain Ther. 2023;12:117–139 (systematic review and meta-analysis): estimates pooled from 17 studies, 10,525 VATS patients (14 studies from China; 3 from South Korea, Italy, and Israel), combining adjusted and unadjusted odds ratios by fixed- or random-effects model depending on between-study heterogeneity for each predictor (model specified in the source per predictor); outcome = CPSP per IASP/ICD-11 definition, with follow-up of ≥2–3 months across contributing studies. The unit for the operative-time continuous predictor is not specified in the source meta-analysis. Notably, the port-number estimate specifically (OR 4.32, 95% CI 3.51–5.13, three ports vs. fewer) is a fixed-effects pooled estimate combining the multivariate analyses of two of the 17 contributing studies, not a single-study finding. [15] Tokuishi et al., Asian J. Endosc. Surg. 2024;17:e13302: single-center cohort of 138 patients undergoing RATS (Japan); multivariate logistic regression; outcome = persistent postoperative pain, defined as analgesic use continuing ≥2 months after surgery. [27] Kawaguchi et al. (J-RATSIG 01), Lung Cancer 2024;196:107961: prospective, multi-institutional Japanese cohort undergoing anatomic lung resection by RATS or VATS; multivariable analysis; primary outcome = NRS pain score ≤3 at postoperative day 30. Tumor location was identified as an independent risk factor in multivariable analysis. [29] Zhang et al., BMC Surg. 2022;22:76: single-center retrospective cohort of 2348 VATS patients (Nanjing, China); multivariate logistic regression; outcome = CPSP at 3 months, assessed by telephone interview. [31] Li & Wu, Front. Surg. 2026;12:1742042: single-center case–control study of 280 patients undergoing minimally invasive thoracic surgery (48 CPSP, 232 non-CPSP; Xinjiang, China); multivariate logistic regression; outcome = CPSP at 3 months. Postoperative shoulder-abduction pain was assessed daily by VAS from postoperative day 1 to day 6. [5,6,16,26,27] (Surgical approach row): port-controlled comparative studies of uniportal VATS versus multiport RATS; see Table 1 and Figure 2 for individual study designs, populations, and timepoints. RCT: Randomized controlled trial; EORTC QLQ-C30: European Organisation for Research and Treatment of Cancer Quality of Life Questionnaire—Core 30; EORTC QLQ-LC13: European Organisation for Research and Treatment of Cancer Quality of Life Questionnaire—Lung Cancer 13; EQ-5D-5L: EuroQol 5-Dimension 5-Level instrument; VAS: Visual analogue scale; NRS: Numeric rating scale; POD: Postoperative day; VATS: Video-assisted thoracoscopic surgery; RATS: Robotic-assisted thoracoscopic surgery.

**Table 3:** Summary of RIOT-Related Evidence.

Study (Ref)	Design/Population	Surgical Platform Comparison	RIOT/Adjuvant-Treatment Endpoint	Pain Directly Measured?	Pain Independently Associated with Endpoint?
Aloia et al., 2014 [7]	Conceptual/methodological paper (multi-cancer surgical oncology, not lung-specific)	None (defines the RIOT metric itself)	Proposes RIOT as a quality metric	No	N/A—foundational definition paper
Salazar et al., 2017 [8]	Retrospective National Cancer Database cohort, lung cancer resection	Not platform-specific	Time to chemotherapy initiation (≤127 days) vs. mortality	No	Not tested—timing itself was not associated with mortality; receipt of any treatment was the stronger determinant
Rabinovich & Martin 2025 [9]	Narrative/perspective review	ERAS framework (not platform-specific)	Conceptual link between ERAS and RIOT	Discussed qualitatively only	Proposed, not empirically tested
Donington & Martin 2019 [32]	Commentary/editorial	None	Calls for RIOT as a standard oncologic-surgery outcome	No	N/A—opinion piece
Nelson et al., 2019 [33]	Retrospective cohort, NSCLC, ERAS vs. non-ERAS pathway	Perioperative pathway (not platform-specific)	Adjuvant chemotherapy completion rate	No (pain not isolated from the ERAS bundle)	ERAS bundle (which includes analgesia) associated with improved completion; pain itself not isolated as an independent variable
Kim et al., 2016 [34]	Narrative/perspective review	ERAS framework (not platform-specific)	Conceptual discussion of complications’ impact on RIOT	Discussed as one contributor within a complications framework	Not empirically tested
Abdelghany et al., 2022 [35]	Retrospective cohort,	RATS vs. open thoracotomy	Adjuvant chemotherapy completion rate (74.3% vs. 75.8%, ns)	No	No
	RATS vs. thoracotomy				

RIOT: Return to Intended Oncologic Treatment. ERAS: Enhanced Recovery After Surgery.

## Data Availability

No new data were created or analyzed in this study. Data sharing is not applicable to this article.

## Supplementary Materials

The following supporting information can be downloaded at: https://www.mdpi.com/article/10.3390/jcm15207843/s1, Supplementary File S1. Representative Literature Search Strategy.
