# Supplementary material for: Rethinking Postoperative Pain in the Era of Robotic Thoracic Surgery: A Surgeon’s Perspective on Functional Recovery and Potential Oncologic Implications
Source: J Clin Med. 2026 Oct 12;15(20):7843. doi: 10.3390/jcm15207843 (PMC13705381; doi:10.3390/jcm15207843)
Supplement: Supplementary file 1 [file jcm-15-07843-s001.zip › jcm-4539467-supplementary.pdf]

Supplementary File S1

Representative Literature Search Strategy

Manuscript: "Rethinking Postoperative Pain in the Era of Robotic Thoracic Surgery: A Surgeon's Perspective on Functional Recovery and Potential Oncologic Implications" — Journal of Clinical Medicine

1. Search Strategy

PubMed/MEDLINE (https://pubmed.ncbi.nlm.nih.gov) was searched from 1 January 2010 to 31 July 2026 (final search date), limited to human studies in the English language (non-English-language studies were considered for inclusion only where an accessible translation existed, per Section 2). The strategy below follows PubMed's standard line-numbered Boolean structure, combining a platform concept block (#1–#3), a pain/injury concept block (#4–#6), an oncologic-treatment concept block (#7–#8), and a study-type filter (#9):

```
#1  "Robotic Surgical Procedures"[MeSH Terms] OR robot-assisted thoracic
surg*[tiab] OR robotic thoracic surg*[tiab] OR robot-assisted
lobectomy[tiab] OR robotic lobectomy[tiab] OR robot-assisted
segmentectomy[tiab] OR RATS[tiab]
#2  "Thoracic Surgery, Video-Assisted"[MeSH Terms] OR video-assisted
thoracoscopic surg*[tiab] OR video-assisted thoracic surg*[tiab] OR
thoracoscopic lobectomy[tiab] OR thoracoscopic segmentectomy[tiab] OR
uniportal[tiab] OR VATS[tiab]
#3  #1 OR #2
#4  "Pain, Postoperative"[MeSH Terms] OR "Chronic Pain"[MeSH Terms] OR
postoperative pain[tiab] OR post-operative pain[tiab] OR chronic
postsurgical pain[tiab] OR chronic post-surgical pain[tiab] OR CPSP[tiab]
OR acute pain[tiab] OR chronic pain[tiab]
#5  "Intercostal Nerves/injuries"[MeSH Terms] OR intercostal nerve
injury[tiab] OR intercostal neuralgia[tiab] OR post-thoracotomy pain
syndrome[tiab] OR neuropathic pain[tiab]
#6  #4 OR #5
#7  Return to Intended Oncologic Treatment[tiab] OR "return to intended
oncologic treatment"[tiab] OR RIOT[tiab]
#8  "Chemotherapy, Adjuvant"[MeSH Terms] OR adjuvant therapy[tiab] OR
adjuvant chemotherapy[tiab] OR time to adjuvant therapy[tiab]
#9  "Randomized Controlled Trial"[Publication Type] OR "Cohort
Studies"[MeSH Terms] OR "Systematic Review"[Publication Type] OR "Meta-
Analysis"[Publication Type] OR randomized[tiab] OR cohort[tiab] OR
propensity[tiab] OR "systematic review"[tiab] OR meta-analysis[tiab]
#10  #3 AND #6
#11  #3 AND (#7 OR #8)
#12  (#10 OR #11) AND #9
#13  #12 AND (2010/01/01:2026/07/31[dp]) AND English[lang] AND
humans[Filter]
```

Final combined search (#13) is the representative string yielding the primary result set screened for this review.

2. Screening and Selection

Titles and abstracts returned by search #13, together with articles identified by hand-searching the reference lists of key trials and recent systematic reviews (Section 2), were screened for relevance by the corresponding author (T.M.) and a second author (R.D.). Full texts of potentially eligible records were then reviewed independently by the same two authors; disagreements were resolved by discussion with the senior author (K.M.). Eligible studies reported acute or chronic pain outcomes after VATS or RATS lobectomy/segmentectomy, or Return to Intended Oncologic Treatment (RIOT)/adjuvant-therapy timing after thoracic surgical resection, and were published between January 2010 and the final search date; case reports and conference abstracts were excluded except where retained for otherwise-unavailable mechanistic or first-in-human data (e.g., ref. 49), identified as such in-text.

Where multiple publications reported outcomes from the same trial or cohort (e.g., the RVlob trial's short-term outcome, quality-of-life, and three-year-survival reports, refs. 3, 17, 18), the report with the longest follow-up or most complete pain-outcome data was treated as the primary source in Table 1; companion reports were cited only for information not otherwise available in the primary report (e.g., long-term survival). No formal risk-of-bias or systematic quality assessment (e.g., Cochrane RoB 2, ROBINS-I, AMSTAR-2) was performed, consistent with this review's non-systematic, narrative design; this is stated as a limitation in Section 10 of the manuscript.
